# High-harmonic spectroscopy of transient two-center interference calculated with time-dependent density-functional theory

**DOI:** 10.1063/1.5111349

**Published:** 2019-07-16

**Authors:** François Mauger, Paul M. Abanador, Timothy D. Scarborough, Timothy T. Gorman, Pierre Agostini, Louis F. DiMauro, Kenneth Lopata, Kenneth J. Schafer, Mette B. Gaarde

**Affiliations:** 1 Department of Physics and Astronomy, Louisiana State University, Baton Rouge, Louisiana 70803, USA; 2 Department of Physics, The Ohio State University, Columbus, Ohio 43210, USA; 3 Department of Chemistry, Louisiana State University, Baton Rouge, Louisiana 70803, USA

## Abstract

We demonstrate high-harmonic spectroscopy in many-electron molecules using time-dependent density-functional theory. We show that a weak attosecond-pulse-train ionization seed that is properly synchronized with the strong driving mid-infrared laser field can produce experimentally relevant high-harmonic generation (HHG) signals, from which we extract both the spectral amplitude and the target-specific phase (group delay). We also show that further processing of the HHG signal can be used to achieve molecular-frame resolution, i.e., to resolve the contributions from rescattering on different sides of an oriented molecule. In this framework, we investigate transient two-center interference in CO_2_ and OCS, and how subcycle polarization effects shape the oriented/aligned angle-resolved spectra.

## I. INTRODUCTION

High-harmonic spectroscopy (HHS) is based on the idea of using the spectral properties of high-harmonic generation (HHG) signals to coherently probe atomic or molecular systems.^1–3^ For instance, it has been used to recover internuclear distances^4–6^ or image molecular wave-functions with tomographic reconstruction algorithms.^7–10^ Conceptually, HHS also provides a built-in temporal dimension with subfemtosecond resolution. This is based on the fact that HHG emission is naturally synchronized with the laser field, and different harmonics are mapped to sublaser-cycle emission times.^11,12^ As an example, HHS has recently been used to measure charge migration in molecules.^13–15^ A complete characterization of increasingly complex molecular structures and dynamics via HHS technique will require multidimensional analyses and access to the full information contained in these HHG signals, and the ability to obtain both amplitude and phase information^16,17^ of (preferably) oriented molecules^18^ will have to become standard.

Theoretically and numerically, the challenges of treating a correlated many-electron system responding to a strong laser field are tremendous and cannot be done without significant approximations. In this context, time-dependent density-functional theory (TDDFT) offers a scalable computational framework for many-active-electron molecules interacting with the laser. In spite of some known drawbacks^19^ (most prominently that an exact exchange-correlation functional is not known), TDDFT has recently been validated against other calculations or experimental results in a number of studies related to ultrafast and strong-field processes, such as high-harmonic spectroscopy,^17^ strong-field ionization,^20–22^ or charge migration.^23^

A difficulty in calculating HHG spectra using first-principles methods is the stark difference between the microscopic-scale field emitted by individual molecules and the measured macroscopic response.^24–26^ The single-molecule response consists of multiple so-called quantum-orbit contributions that give rise to broad, unresolved spectral features.^27^ The macroscopic response is dominated by phase-matched radiation originating from the shortest quantum-orbit contribution, which is reasonably well resolved in the temporal, spectral, and spatial domains. HHS is built upon the intrinsic coherence of HHG spectra, where the same electron that was first ionized returns and probes its parent cation.^28,29^ Meaningful information about the target's structure and dynamics can therefore be extracted only from harmonic emission originating from a single quantum orbit contribution. Numerically, macroscopic effects can, in principle, be accounted for by incorporating the single-molecule response into a wave-equation solver throughout the generating gas medium, as is routinely done for single-active-electron systems.^30,31^ However, the individual computation cost of one TDDFT-HHG calculation still makes this approach prohibitive for more complex systems.

In this paper, we use TDDFT to explore HHS in two linear molecules at mid-infrared (MIR) wavelengths: the symmetric (nonpolar) CO_2_ and the asymmetric (polar) OCS molecules. We calculate the harmonic spectral amplitude and phase in the vicinity of a two-center interference (TCI) minimum in each molecule, as a function of the relative angle between the molecular axis and the linearly polarized laser field—see Ref. 17 for more details on TCI and direct comparisons with experimental results. We show that we can reliably select the contribution from the short quantum orbits to the harmonic spectrum by combining the MIR laser field with an attosecond pulse train (APT) that coincides with the ionization time of the short quantum orbit in each half-cycle of the field. As demonstrated in helium atoms,^32,33^ this “ionization seed” leads to a harmonic spectrum dominated by the short-orbit contribution. We show that this yields TDDFT-calculated spectral amplitudes and phases which are sufficiently well-resolved to recognize the characteristic angle-dependence of the TCI minimum.^17^ We also find that by selecting the short-quantum-orbit contribution from a single laser half-cycle, we naturally obtain molecular-frame information about the HHG process since we can discriminate between rescattering from the two sides of an asymmetric molecule. As an example, we show that the harmonic spectrum is substantially different when the returning electron rescatters on the O side relative to the S side of OCS, and we interpret this in terms of the instantaneous field-induced redistribution of the electron density in the molecular core. In particular, we show that when rescattering on the electron-heavy S side, the laser field rebalances the distribution of charge density, so that the two centers are more even than in the undressed molecules, and this leads to a clear TCI minimum in the harmonic spectrum.

This paper is organized as follows. Section II describes our theoretical and numerical approach, detailing the TDDFT and ionization-seed numerical methods as well as the HHS analysis. In this section, we also show some technical results demonstrating the efficacy of the ionization seed. Section III presents angularly resolved HHS investigations in the molecular-frame picture as discussed above, showing the difference between rescattering from the S or O side of the OCS molecule, as well as their interplay when forming aligned-only spectra. Then, in Sec. IV, we interpret the observed spectral features in terms of transient TCI processes, where two localized density components of the target's electronic structure lead to harmonic emission which interferes destructively. We show that such TCI features provide a good landmark to probe subcycle density dynamics in the compound. Finally, Sec. V concludes our analysis and discusses possible application perspectives for the study of charge migration in molecular targets.

Unless otherwise stated, atomic units are used throughout the paper. We also define the angle *θ* between the oriented molecular axis of CO_2_/OCS and the laser-field polarization direction.

## II. METHODS

### A. Time-dependent density-functional theory simulations

For the TDDFT computations, we use a local-density approximation (LDA) exchange-correlation potential^34–37^ with average-density self-interaction correction^38^ (ADSIC), as implemented in OCTOPUS.^39,40^ The self-interaction correction ensures that the asymptotic (long-range) behavior of the potential is Coulombic, as is necessary for proper HHG spectra.^41–43^ The Kohn-Sham equations are solved on a Cartesian grid with a spacing of 0.4 a.u. in all directions and with the molecule centered in the simulation box. The dimensions of the box are 195 a.u. in the direction parallel to the laser polarization and 30 a.u. in the perpendicular directions. The temporal step for all time-dependent computations is 0.05 a.u. and we have checked the convergence of reported spectra for all computational parameters. For reference, we find numerical ionization potentials (energy of the highest-occupied molecular orbital) of *I_p_* = 14.55 eV for CO_2_ and *I_p_* = 11.67 eV for OCS, as compared to the experimental values of 13.77 eV and 11.17 eV, respectively.^44^

**FIG. 1.**
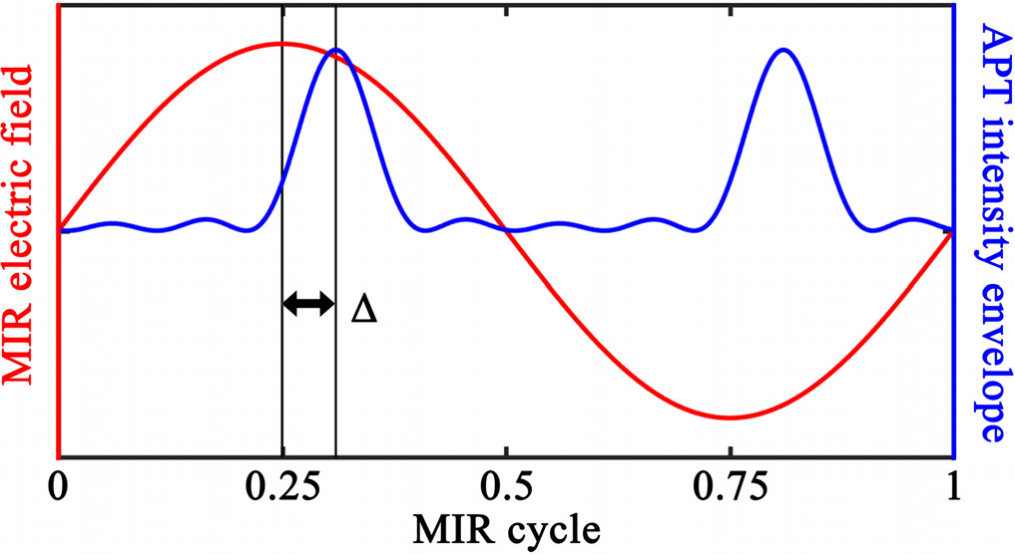
Illustration of the laser fields used to select short-quantum-orbit contributions to harmonic spectra in TDDFT computations. The weak ionization-seed APT is synchronized with the strong driving MIR field. The delay Δ between the APT and MIR is selected to get the best enhancement of short-contributions only (see Sec. II C).

As discussed in the Introduction, the laser field is composed of a strong driving MIR plus a weak ionization-seed APT, synchronized with the MIR—see Fig. 1. For the MIR, we use a linearly polarized laser field with a wavelength of 1500 nm and an intensity of 60 TW/cm^2^, which leads to a harmonic plateau that extends to 50–60 eV in the two molecules. This means that the TCI minimum is within the plateau range for angles up to about 45°. The field envelope is ramped up with a $\;{\text{sin}}^{2}$ shape during the first two cycles of the pulse, and then kept constant. The harmonic spectrum is calculated from the constant-intensity part of the pulse, as described in detail in Sec. II B.

The APT ionization seed is composed of 5 MIR-odd-harmonics with energies close to the ionization threshold, with harmonics 9 through 17 in CO_2_, and 7 through 15 in OCS. The APT intensity and the subcycle delay relative to the MIR electric field are chosen such that the HHG yield is dominated by the contribution from electrons that are one-photon-ionized by the seed:^32^ The envelope of the APT is matched to that of the MIR, and its peak intensity is either 2% (CO_2_) or 1% (OCS) of the MIR. The timing of the APT ionization seed is chosen such that it coincides with the time during the MIR cycle at which the short orbits are initiated. This means that the APT should be centered shortly after the peaks of the MIR field, as illustrated in Fig. 1. For the calculations shown in this paper, we have used delays Δ between 0.06 and 0.08 MIR cycles. More details about the timing of the APT are provided in Sec. II C.

### B. High-harmonic spectroscopy

#### 1. Spectral yield

The harmonic spectral properties are calculated from the TDDFT time-dependent dipole acceleration signal a(t,θ), which has components that are both parallel and perpendicular to the laser polarization. The spectral intensity is the incoherent sum of the parallel and perpendicular contributions

$${\left|\text{HHG}\left(\nu ,\theta \right)\right|}^{2}={\left|F[W(t)a_{∥}(t,\theta )]\right|}^{2}+{\left|F[W(t)a_{⊥}(t,\theta )]\right|}^{2},$$ (1)

where *ν* is the frequency, F denotes the Fourier transform, and *W*(*t*) is a time-selection window, which is shaped like $\;{\text{cos}}^{4}$. *W*(*t*) allows us to select the time-dependent response from one or multiple half-cycles of the laser field. Selecting a single half-laser-cycle allows us to distinguish the harmonic spectrum by orientation of an asymmetric molecule, i.e., we expect to see a difference between molecules of opposite orientations (see Sec. III).

**FIG. 2.**
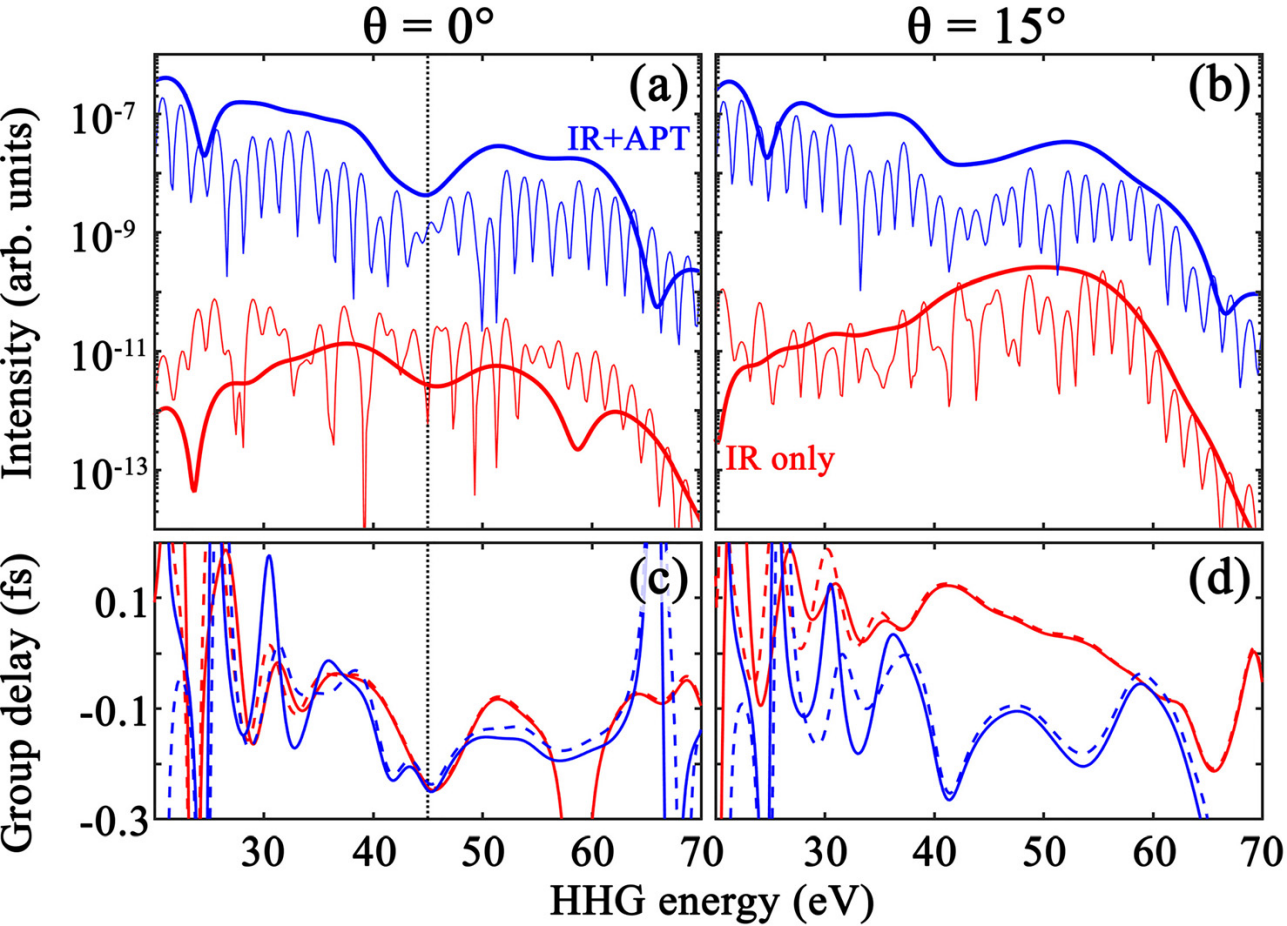
HHS analysis of TDDFT simulations in CO_2_ for 0° (left panels) and 15° (right) alignments. Panels (a) and (b) compare the spectral intensity of Eq. (1) with the MIR-only and APT-ionization-seed (see labels). Single sets of short orbits (thick curves) are selected with a window *W*(*t*) spanning 2.05-to-2.55 laser-cycles, and full spectra (thin) use a window between 2.05 and 4.05 cycles. Panels (c) and (d) show the single-short-orbit target-specific group delay of Eq. (4) using a one-dimensional (dashed curves) and a two-dimensional (solid) reference. The driving MIR has 1500-nm wavelength and 60-TW/cm^2^ intensity; the parameters of the APT seed are specified in Sec. II A. The vertical dotted line in (a) and (c) labels 45 eV.

Figures 2(a) and 2(b) show the spectral yield in the symmetric molecule CO_2_, for alignment angles of (a) θ=0° and (b) θ=15°, generated by the MIR alone (lower red curves) and by the MIR+APT (upper blue curves). The thick and thin curves, in each case, correspond to using two different window functions, which select either a single half-cycle (from 2.05 to 2.55 cycles, thick lines) or four half-cycles (from 2.05 to 4.05 cycles, thin lines), respectively, of the time-dependent response. The figure demonstrates several important points:

First, at 0°, the TCI is clearly visible with a local minimum in the spectral intensity around 45 eV (vertical dotted line). Physically, the TCI minimum can be understood as a destructive interference between the scattering contributions from the two ends of the molecule^45–47^ (see Sec. IV). In this picture, the energy location of the minimum is related to the effective distance between the two centers seen by the (re)scattering electron.^48,49^ As such, the position of the TCI is expected to shift to higher energies as *θ* is increased away from the parallel orientation. This angle dependence has been confirmed experimentally for CO_2_, although it is not as fast as predicted by a plane wave rescattering electronic-wave-function.^17^

Second, Figs. 2(a) and 2(b) illustrate the effect of the APT on the harmonic spectrum: (i) for the seed intensity we consider here, the APT clearly dominates the ionization step and leads to an HHG spectrum which is several orders of magnitude stronger than that of the MIR alone, (ii) the APT does not substantially influence the position of the cutoff energy, and (iii) the APT-initiated spectrum is much cleaner than the MIR-alone spectrum, with odd harmonics clearly separated from each other (thin curves). All of these points are consistent with the findings on helium atoms in Ref. 32, and can be understood from the three-step picture of HHG in which the spectrum is the product of an ionization probability, a rescattering wave packet, and a recombination dipole moment.^50–52^ The APT can clearly enhance the ionization step by substituting tunnel ionization with one-photon ionization, and its timing is such that it predominately launches electrons on the short quantum orbit (see more details below). However, it has a negligible influence on the continuum dynamics, which is controlled by the MIR field, and on the recombination probability, which is specific to the molecule. In addition, the use of the short time-selection window (thick curves) further eliminates interferences between dynamics initiated in consecutive half-cycles and isolates the contribution from a single half-cycle, while not changing the overall spectral shape. Typically, the cleanest signal is obtained during the first half laser cycle of the plateau, which combines the effects of the APT seed and the fact that a single set of recolliding orbits has been effectively generated by the MIR thus far. Finally, the TCI minimum is much clearer in the APT-seeded spectra; at 15° the minimum is not even visible in the MIR-alone spectrum, and at 0° it is weaker than in the seeded spectrum.

**FIG. 3.**
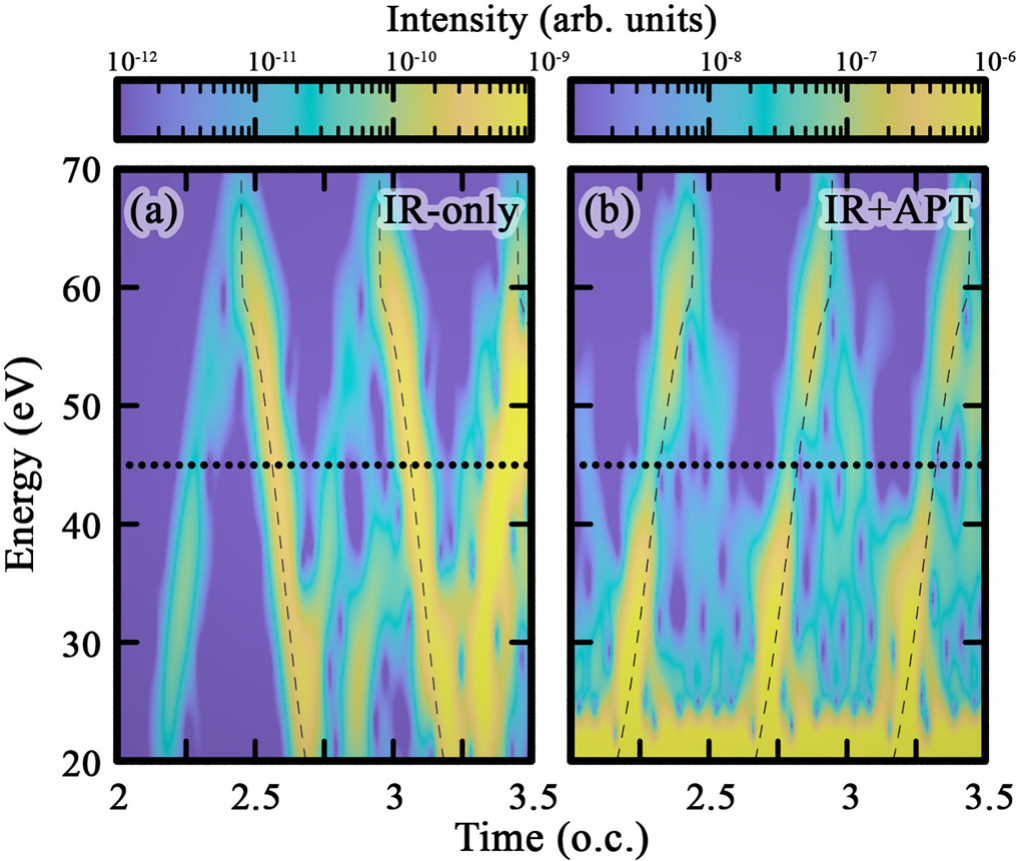
Spectrograms of TDDFT simulations in CO_2_ for 0° with the (a) MIR only and (b) APT-ionization seed. On each panel, dashed curves label strong-field-approximation (a) long- and (b) short-orbit contributions.^27^ The horizontal dotted line labels 45 eV. Parameters are the same as in Fig. 2(a) (see also Sec. II A).

Figure 3 further illustrates the effect of the ionization seed. It shows the time-frequency representation (spectrogram) of the harmonic emission in the first three half-cycles of constant MIR intensity, (a) without and (b) with the APT seed. The spectrogram is computed from Eq. (1), using a $\;{\text{cos}}^{4}$ sliding window with 1-fs duration. Again, Fig. 3(a) shows how, in the absence of the APT, both the short and longer quantum orbits contribute to the HHS signal and the long orbit dominates. The TCI minimum is only weakly visible in the short-orbit branch. In contrast, the APT-seeded spectrogram in (b) is strongly dominated by the short-orbit contribution, which has a clear TCI minimum. It is also worth noting that in the APT-seeded case, in the low-energy part, there is a substantial amount of emission which does not seem to follow any particular orbit contribution. This is most likely caused by electrons from lower-lying orbitals that can be ionized by the broad-bandwidth APT, and then contribute to the continuum and rescattering dynamics. We will come back to this issue in Sec. II C.

Figure 3 also illustrates why the time-selection window in combination with the APT seed works well to select a single set of short quantum orbits: each set of ionization-seed-enhanced short orbits is temporally separated, roughly between successive zeros of the laser electric field. We also note that the time-frequency map has half-cycle periodicity, which again supports the idea that the ionization seed enhances the short orbit signal without triggering longer-time scale processes.

#### 2. Group delay

We also extract phase information from the TDDFT calculations and calculate the target-specific group delay (GD). Experimentally, the GD is extracted from interferometric measurements,^53,54^ and it is related to the spectral phase by a derivative. The target-specific GD is the contribution to the GD from rescattering alone, and it is calculated after normalizing the acceleration spectrum by that of a companion atom with an identical *I_p._*^17,55^ This normalization can be understood in the framework of the three-step model and formalized in the quantitative rescattering (QRS) approach, where HHG signals are factorized in the frequency (*ν*) domain^50–52^

HHG(ν;θ)=Ion.(θ)×Prop.(ν)×Scatt.(ν;θ). (2)

The three pieces correspond to the contributions from the angle-dependent ionization yield (Ion.), a generic electron wave packet associated with the laser-driven propagation of the ionized electron (Prop.), and the target-specific and angle-dependent (re)scattering (Scatt.). In this framework, a one-electron spherically symmetric atom with the same *I_p_* would give rise to the same generic wave packet with a nearly flat scattering phase, and can therefore be used as a reference. The target-specific group delay of the molecule can then be calculated as

$$GD_{∥/⊥}\left(\theta ;\nu \right)\approx -\frac{\partial }{\partial \nu }\text{Arg}\left(\frac{F\left[Wa_{∥/⊥}\left(\theta \right)\right]\left(\nu \right)}{F\left[Wa_{\text{ref}}\right]\left(\nu \right)}\right),$$ (3)

where $a_{\text{ref}}\left(t\right)$ is the acceleration signal from the reference atom, subjected to the same MIR+APT laser field, and calculated by numerical solution of the time-dependent Schrödinger equation. At the very least, the companion-atom normalization provides a common reference to compare features in the GD, e.g., as the orientation/alignment angle is varied. Finally, the total GD is obtained by weighing the contributions from the parallel and perpendicular directions with their respective intensities

$$\mathrm{GD}=\frac{{\left|F\left[Wa_{∥}\right]\right|}^{2}GD_{∥}+{\left|F\left[Wa_{⊥}\right]\right|}^{2}GD_{⊥}}{{\left|F\left[Wa_{∥}\right]\right|}^{2}+{\left|F\left[Wa_{⊥}\right]\right|}^{2}}.$$ (4)

The weighing above ensures that the results of Eq. (4) are unchanged by a rotation of the simulation domain with a fixed angle *θ*.

The CO_2_-specific GD for two different alignment angles is shown in Figs. 2(c) and 2(d), for both the MIR-alone (red) and MIR+APT (blue) cases. At 0°, both the MIR-alone and the APT-seeded GDs show a minimum at the position of the TCI minimum. One would expect to see such a minimum (or maximum) in the GD signal around the TCI, as it corresponds to a phase shift of near ±*π* associated with the destructive interference seen in the amplitude. For the 15° case, the MIR-alone GD, again, shows no discernible feature near the minimum. Figures 2(c) and 2(d) also show a comparison of results using a one-dimensional (dashed curves) and a two-dimensional (continuous) reference atom in Eq. (4). The close similarity between the two makes us confident in our method for extracting target-specific group delays.

Compared to the amplitude part of HHG signals, meaningful target-specific GDs are much more challenging to extract. Part of this is due to the fact that the phase typically carries most of the information (for example, the phase is the basis for tomographic reconstruction algorithms^56^). Additionally, the generic part of the GD (the attochirp) usually dominates over the target-specific part that is of interest to us. Finally, despite our best efforts to minimize its effects, the addition of the APT field contributes to the overall HHG phase, and thus the GD, possibly including at the ionization step. The reference normalization of Eq. (3) plays an important role in removing (some of) these systematic contributions. Because the reference is computed with an identical total field, including the APT, we expect the effects of the ionization seed to be mitigated in the target-specific GD.

The limitations of target-specific group delay extractions are most visible in the low-energy part of the spectrum, roughly below 35 eV in Figs. 2(c) and 2(d), where the extracted GD oscillates rapidly and is unreliable. This could be caused by interference with lower-lying orbital contributions that are made accessible by the broad-band APT. Also, note that lower frequencies typically span longer times (longer periods), and are therefore more prone to interference or noise. Similarly, in the cut-off region beyond about 60 eV in the figure, the GD becomes unreliable because the amplitude vanishes and there is no signal to extract the phase information from. On the other hand, the close similarity of the results using one-dimensional (dashed curves) and two-dimensional (continuous) references highlights the robustness of our method for extracting target-specific GDs. Whereas challenging, experimental campaigns have demonstrated the crucial role of phase information in completing a full characterization of TCI and other structural features.^17,55,57–59^

### C. Timing of the APT ionization seed

**FIG. 4.**
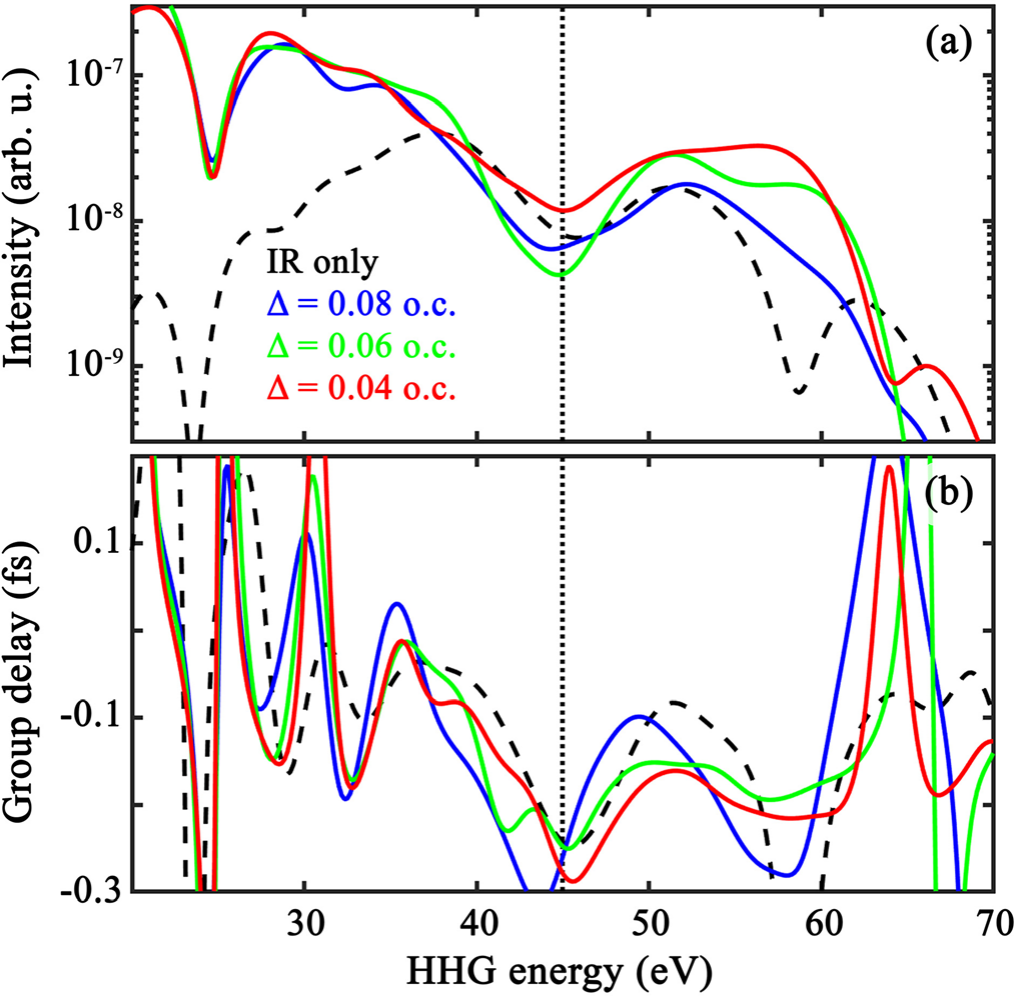
Comparison of the (a) spectral intensity of Eq. (1) and (b) target-specific group delay of Eq. (4) with varying APT-MIR timing—see legend and Fig. 1 for the definition of Δ. Here, we compare results in CO_2_ at 0° alignment from the first set of short orbits in the plateau. Other parameters are the same as in Fig. 2(a) (see also Sec. II A).

We mentioned above that the APT delay should be optimized for each system. In Fig. 4, we compare the (a) spectral intensity and (b) target-specific GD in CO_2_ using different MIR-APT delays (see legend). It shows that, while qualitatively similar, the specifics of both signals are influenced by the delay, and the GD is the most sensitive to it. For Δ = 0.08 MIR cycles (blue), the low-energy part of the spectrum is preferentially enhanced leading to a less well-defined cut-off in spectral intensity. On the other hand, the spectrum for Δ = 0.04 MIR cycles has the best-defined cut-off, but a less sharp TCI minimum. This suggests that optimal delays have a slight dependence on the HHG frequency, which is compatible with selective subcycle enhancement of ionization by the APT seed. Here, to avoid artifacts associated with changing the delay while other parameters are varied (*θ*), we select a fixed delay that gives the best spectra over all angles. For CO_2_, we find that this best delay corresponds to 0.06 optical cycles after the MIR peaks (and 0.08 cycles for OCS).

### D. Aligned signal

**FIG. 5.**
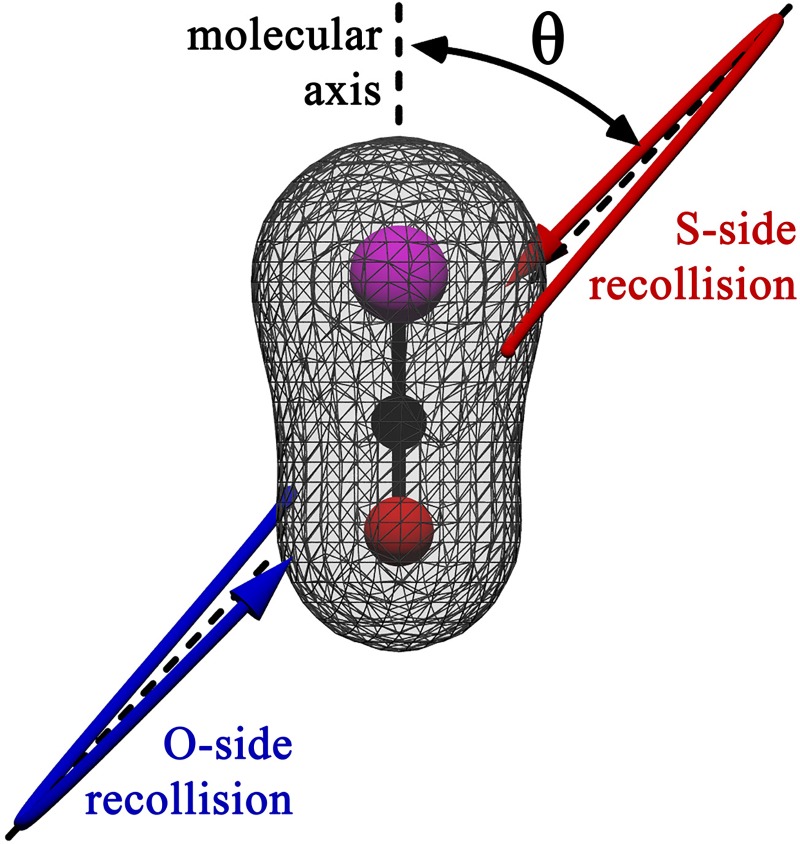
Illustration of molecular-frame analysis provided by half-laser-cycle-resolved HHS. By considering the short orbit contributions from individual half-cycles, we resolve the individual contributions to HHS spectra from ionization and recollision from the S (upward red arrow) and O (downward blue) sides of OCS.

For asymmetric targets like OCS, the angle *θ* is defined between the oriented molecular and laser polarization axes. As illustrated in Fig. 5, this means discriminating between “head” (here S) and “tail” (O) molecular orientations. Experimental measurements, however, may achieve alignment only, i.e., with a macroscopic mixture of molecules with opposite head-tail orientations. Even in instances where orientation is achieved experimentally, the degree of orientation is often very small.^60–63^ Accordingly, we also compute spectral properties of aligned molecules by substituting a coherent average of the acceleration signals in opposite-orientation directions

$$a_{∥/⊥}^{\text{aligned}}\left(\theta \right)=\frac{a_{∥/⊥}\left(\theta \right)+a_{∥/⊥}\left(\theta +180{}^{\circ}\right)}{2},$$ (5)

in Eqs. (1)–(4). Alternatively, assuming that the APT ionization seed generates a new set of short orbits without longer time scale effects, one may use the time-inversion symmetry of the MIR+APT laser

$$a_{∥/⊥}\left(t,\theta +180{}^{\circ}\right)=a_{∥/⊥}\left(t+\frac{\pi }{\omega },\theta \right).$$ (6)

In practice, longer time scale effects are never fully suppressed, and Eq. (6) is only ever an approximate way to obtain the signal from the opposite orientation. The approximate time-inversion symmetry, however, can be used as a quality test for the ionization seed, as discussed in the following section.

## III. MOLECULAR-FRAME HARMONIC SPECTROSCOPY WITH TDDFT

We next move on to molecular-frame HHS of the asymmetric molecule OCS—see Fig. 5. As discussed above, the selection of a single half-cycle of the time-dependent response allows us to compare the individual contributions from recolliding orbits from the S (upward red arrow) and O (downward blue) sides of the compound. The comparison of S- and O-scattering sides can therefore be done either by rotating the system (θ↦θ+180°) or comparing the results from two consecutive half laser-cycles.

**FIG. 6.**
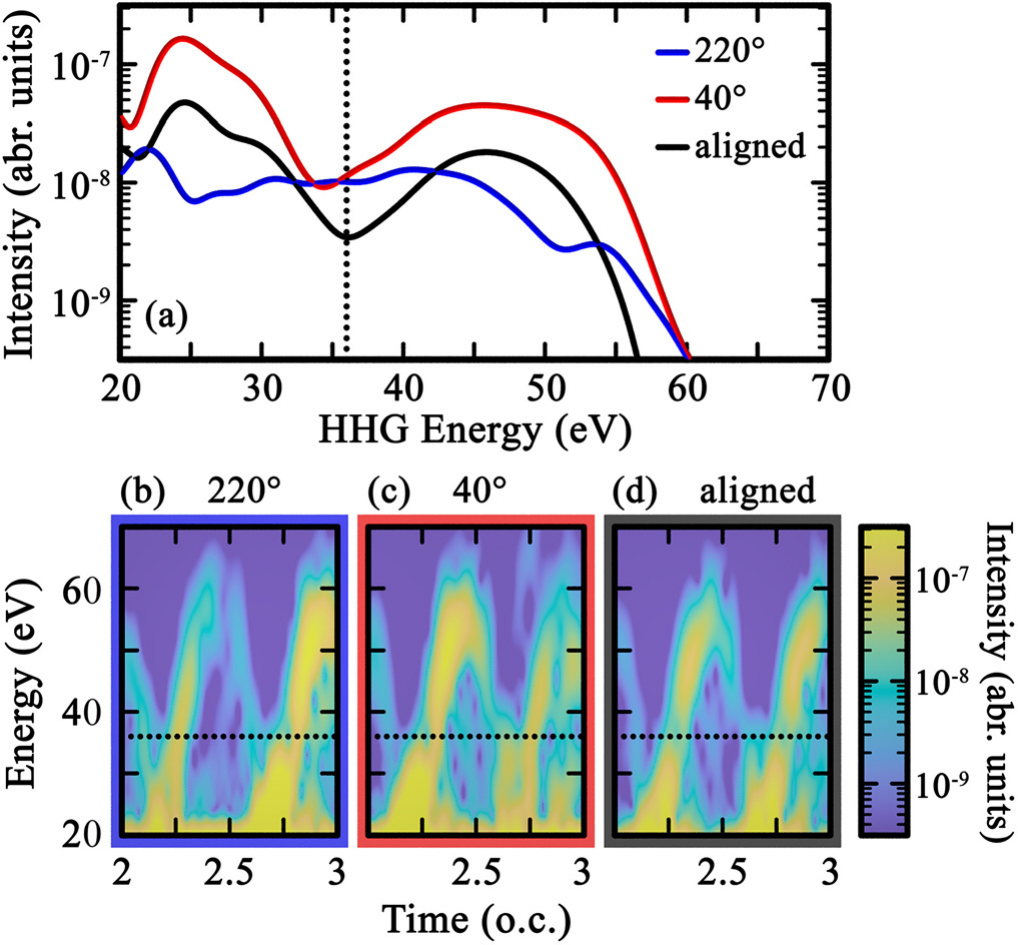
Molecular-frame harmonic spectroscopy in OCS. Panel (a) compares the spectral intensity for oriented molecules with recollision from the O side (220°, blue curve) and the S side (40°, red), and the aligned target as defined by Eq. (5) (black)—see also Fig. 5. For each curve, the corresponding spectrograms are displayed in panels (b)–(d), respectively. The driving MIR has 1500-nm wavelength and 60-TW/cm^2^ intensity; the parameters of the APT seed are specified in Sec. II A. Dotted lines label 36 eV.

Both of these types of comparisons are illustrated in Fig. 6. First, panel (a) shows the spectral yield from opposite orientations of OCS shown in Fig. 5, as well as that of the aligned signal. It is clear from the figure that there is a stark difference between recolliding on the O and S sides: recolliding on the S side leads to an overall much larger yield as well as a clear TCI minimum near 35 eV, which is absent when recolliding on the O side. The figure also shows the alignment-only signal (see Sec. II D). Compared to its two oriented components, we see that the OCS aligned spectral intensity does not correspond to the intensity-average between its two orientation contributions. This is best visible in the energy shift of the aligned TCI as compared to 40°. It highlights the interplay and interference between the two orientations' signals that reshape—and sometimes even occlude—the molecular-frame TCI.^49^

Second, the difference between the two orientations is also illustrated in the spectrograms shown in Figs. 6(b) and 6(c). For each orientation, the consecutive half-cycles of the spectrogram display different behaviors, corresponding to the direction of the field pointing either toward the O or the S end of the molecule at the time of recollision, and the behavior in the two half-cycles is switched when the orientation of the molecule is changed by 180°. With the aligned signal, in panel (d), we recover the expected half-laser-cycle periodicity, and a slight shift in energy of the TCI minimum is observed.

**FIG. 7.**
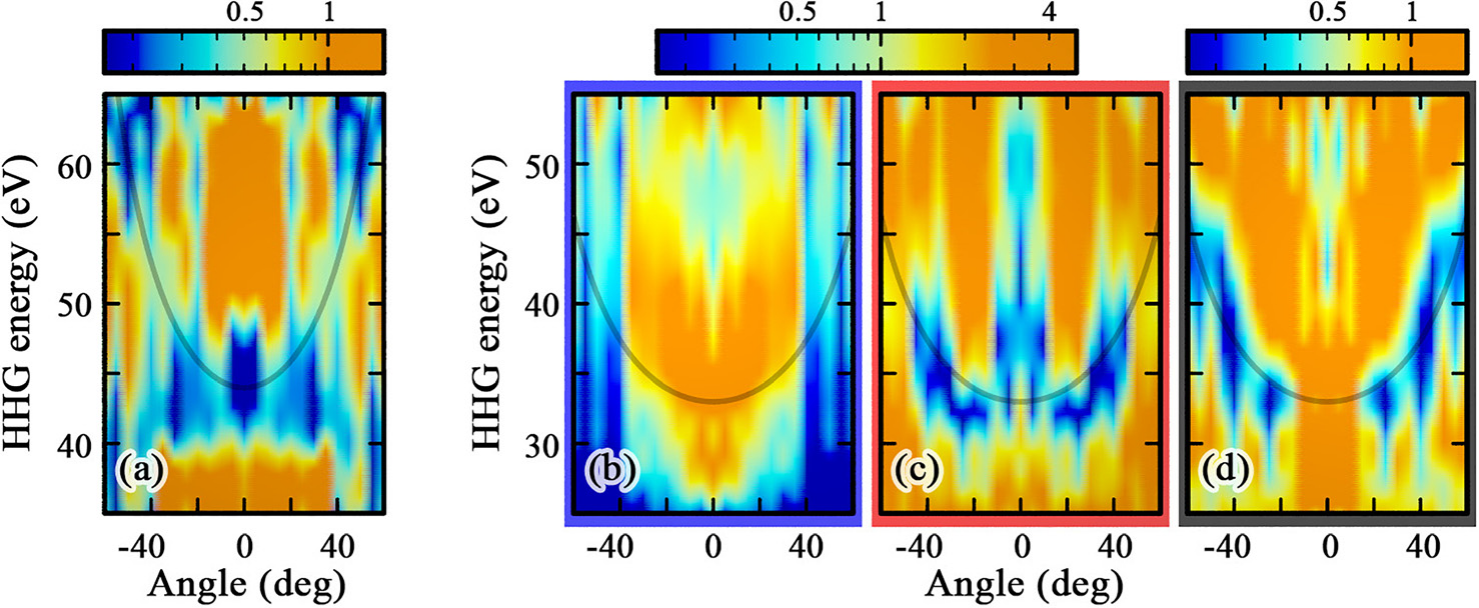
Normalized spectral intensities in (a) CO_2_ and (b)–(d) OCS—oriented molecular-frame scattering from O and S, and aligned, respectively—as functions of the angle *θ*. Note that the color maps in panels (a) and (d) and (b) and (c) span different ranges of intensities. For each compound, the normalization corresponds to a smoothed incoherent average of the aligned signal and is used to reveal features at and beyond the cutoff. In each panel, the thin black curve is set to $I_{p}+\alpha /{\left|\;\text{cos}\;\theta \right|}^{\beta }$, where *I_p_* is the target's ionization potential, as a visual guide for the TCI location.^17^ For CO_2_ (respectively OCS), we set $\alpha =44\;\;\text{eV}-I_{p}$ (respectively $\alpha =33\;\;\text{eV}-I_{p}$) and *β* = 1 (respectively 0.7). Laser parameters are the same as in Figs. 2 and 6 (see also Sec. II A).

In Fig. 7, we perform a systematic angle scan and compare the spectral intensities as functions of *θ* for both (a) CO_2_ and (b)–(d) OCS. In panel (a), we clearly see the TCI shift to higher energies as the alignment is moved away from parallel—see the thin black guiding curve. From the oriented molecular-frame contributions, panels (b)–(c), we see that while a TCI minimum is visible in the S-side-scattering component around 0°, its signal is masked in the component of the O-side, leading to the absence of a TCI in the aligned-response for small angles [panel (d)]. For aligned OCS, the minimum is only apparent for angles |θ|≥20°. More generally, a detailed comparison of the three spectral-intensity maps around the thin black guiding curve once again highlights the subtle interplay between molecular-frame components in forming the aligned signal. In Ref. 17, we found a good qualitative agreement between TCI measurements in aligned CO_2_/OCS and our TDDFT computations.^64^

To better understand the origin of differences between molecular-frame signals recolliding from opposite sides of a molecule, we turn back to the HHG spectrum factorization of Eq. (2). The ionization piece (Ion.) does not depend on the HHG energy and can only account for the overall relative strength of one molecular-frame orientation vs the other. Our finding here that the S-side-recollision spectra have a higher overall yield is consistent with recent TDDFT calculations of orientation-dependent ionization yields.^22^ The propagation piece (Prop.) is generic across atomic and molecular species and does not contain any alignment/orientation dependence and cannot explain the different behavior of the O-side and S-side spectra. This leaves the scattering component (Scatt.) as the only contribution that can influence the location and the depth of the TCI minimum.

The scattering term in Eq. (2) is often described as the field-free cross-section^52^

$$\left\langle \psi_{b}\left|\hat{d}\right|\psi_{c}\left(\nu ;\theta \right)\right\rangle ,$$ (7)

where *ψ_b_* is the bound-component part of the wave function—or an orbital representation in DFT frameworks—and $\hat{d}$ is the dipole/velocity/acceleration operator. *ψ_c_* represents a normalized continuum state with the momentum mapped to the HHG emission energy *ν* and the rescattering angle *θ*. The continuum piece can be approximated by plane wave, Coulomb-corrected, or exact continuum states, with various degrees of accuracy.^51,52,65–67^ In this field-free case, the difference between scattering from the S and O sides of the molecule (for any angle) is simply a sign-change of the phase of *ψ_c_*, and therefore also cannot account for the intensity difference between *θ* and θ+180°. However, the consideration of the field-free scattering cross section is clearly an approximation since the laser field is present during the rescattering, as we discuss in more detail in the remainder of the paper.

In OCS, independent TDDFT simulations, supported by matching ionization measurements in the near-infrared,^22^ have revealed the importance of transient polarization effects. The electronic density distribution around the molecular centers is reshaped by the instantaneous laser electric field.^68^ In the context of HHG, given that short trajectories return roughly between successive zeros of that electric field, it means that plateau harmonics are emitted around peaks of the field when such polarization effects are most prominent. In the context of factorization in Eq. (2), this suggests that the scattering component should also account for the field dressing

$$\text{Scatt}.\left(\nu ;\theta ,{\vec{E}}_{\nu }\right),$$

where ${\vec{E}}_{\nu }$ is the instantaneous electric field at the time of recollision for the quantum orbit leading to the HHG energy *ν*. More practically, the scattering matrix element (7) becomes

$$\left\langle \psi_{b}\left({\vec{E}}_{\nu }\right)\left|\hat{d}\right|\psi_{c}\left(\nu ;\theta ,{\vec{E}}_{\nu }\right)\right\rangle ,$$ (8)

where the bound component, and eventually the continuum too, are dressed by the instantaneous field.^47^ In Sec. IV, we focus on the bound part and revisit our molecular-frame spectra in the context of transient TCI, driven by subcycle polarization effects.

## IV. TRANSIENT TWO-CENTER INTERFERENCE

Formally, the two-center interference picture of OCS corresponds to a decomposition of the bound part of the wave packet between the two components associated with the S and O ends of the molecule: $\psi_{b}=\psi_{b}^{S}+\psi_{b}^{O}$, e.g., rooted in a linear decomposition of the atomic orbital of the bound state. Injecting this ansatz in the scattering matrix element [or its dressed version (8)], one gets

$$\left\langle \psi_{b}\left|\hat{d}\right|\psi_{c}\right\rangle =\underset{\text{Scatt}._{S}}{\underset{︸}{\left\langle \psi_{b}^{S}\left|\hat{d}\right|\psi_{c}\right\rangle }}+\underset{\text{Scatt}._{O}}{\underset{︸}{\left\langle \psi_{b}^{O}\left|\hat{d}\right|\psi_{c}\right\rangle }}.$$ (9)

In this picture, the problem is mapped to the interference between the two “localized” scattering terms Scatt._*S*__∕__*O*_, based on their relative amplitude and phase. Assuming that the two contributions are approximately out of phase by *π* (a very good approximation in the field-free case), their relative amplitude will determine the depth of the minimum. Intuitively, the amplitude part is related to the amount of electron density localized around each thus-defined center. In this section, we revisit our TDDFT results based on that idea and the transient dressing imposed by the instantaneous driving MIR laser field.

**FIG. 8.**
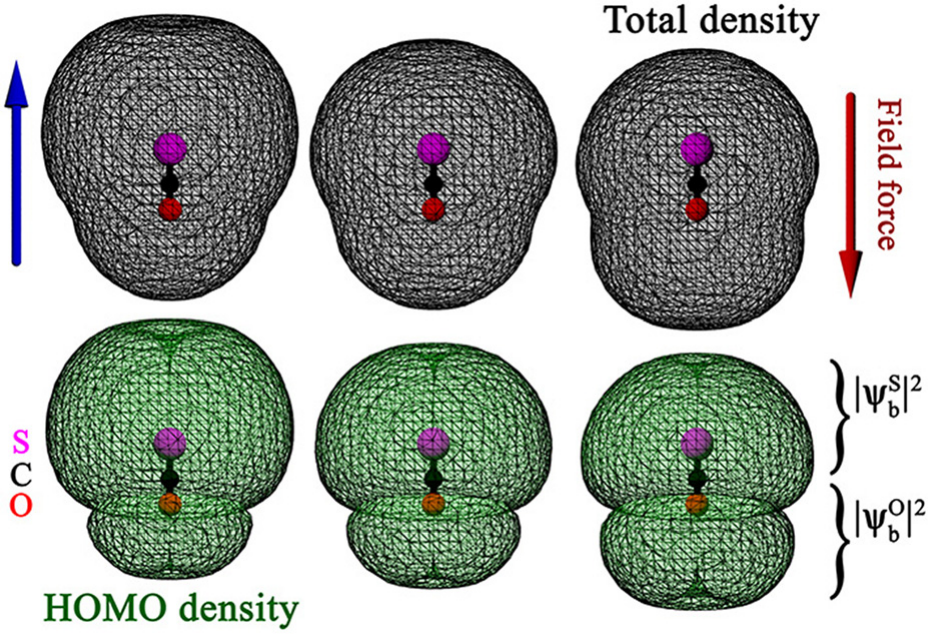
Isosurface of the electron density in the total and joint degenerate highest-occupied molecular orbital (HOMO) densities of OCS subjected to a static electric field with polarization parallel to the molecular axis. From left to right, the field strength is that of the maximum amplitude of an AC field with 20 TW/cm^2^ pointing from S toward O, no field, and 20 TW/cm^2^ pointing from O toward S (see side arrows for the field-induced force direction). All isosurfaces are set to 10^−8^ electrons/a.u.^3^ $\psi_{b}^{S/O}$ illustrates the conceptual decomposition of the wave function bound component between the S and O sides of the molecule—see Eq. (9).

To better understand the effects of the transient MIR dressing on the bound density, we independently compute static-field dressed orbitals in OCS. Here, computations are also performed with OCTOPUS^39,40^ with the same parameters as described in Sec. II A except for the box, which for this time-independent calculation is reduced to 20 a.u. in all directions. The results for θ=0° are shown in Fig. 8. The figure compares the total density (top row) and the density from the degenerate highest-occupied molecular orbitals (HOMO, bottom). From left to right, it shows the density reshaping imposed by the electric field when the induced force points from O-to-S (left, with an arrow indicating the force), vanishes (middle), and S-to-O (right). Overall, and as expected, we see that the field-free imbalance, biased toward the S-side, is magnified when the force is directed O-to-S (compare the central and left columns, respectively). Alternatively, when the force direction is reversed, its action tends to rebalance the density components around the two centers (central and right columns). For aligned signals, the energies of the dressed orbitals (not shown) are also important. Indeed, an orientation-dependent Stark shift means that orbit contributions scattering from the S and O sides do not accumulate the same amount of phase. This extra bit of phase can also contribute to shifting of the observed TCI minimum.^49^

We now combine the dressed-density picture of Fig. 8 with molecular-frame HHG spectra of Figs. 7(b) and 7(c) and find a consistent agreement with the localized-density qualitative TCI picture discussed above: First, when the short trajectories recollide from the O side—panel (b)—the laser-induced force points from O to S at the time of recollision and magnifies the field-free S-to-O imbalance. Accordingly, irrespective of the orientation angle, no TCI is observed. Second, in the reversed configuration, where recollision happens from the S side—panel (c)—the laser force counteracts the field-free imbalance and the TCI is apparent for some angles, roughly between 15° and 45° (see also the thin black guiding curve). Third, the extinction of the TCI minimum for *θ* larger than about 45° could be explained by a reduction of polarization effects, due to: (i) orientation. From Ref. 22, we know that it is the component of the electric field parallel to the molecular axis that is predominantly responsible for “moving” electron densities between the two molecular centers. (ii) The TCI location in energy. For larger angles, the TCI moves toward and beyond the cutoff energy, and is thus being probed by recolliding electrons that return closer to the zero of the electric field. Both effects participate in reducing the dressing-induced rebalancing between the two centers at larger angles. Finally, and as per similar arguments, we wonder if the dressing around parallel orientation, where the expected TCI happens around the laser-peak-field amplitude, overcompensates the field-free imbalance and also ends up “destroying” the minimum (|θ|≲15°).

Overall, we see that multidimensional HHS around TCIs offer a window to observe subcycle dynamics in molecules, between the two centers. The energy of the TCI minimum serves as the ultrafast clock, through mapping with the time of recollision.^11,12^ The sharpness of the spectral amplitude minimum measures the relative distribution of electron density between the two centers. The (sign of the) associated GD feature informs on the “orientation” of that distribution. For instance, a symmetry inversion of the bound component $\psi_{b}\left(x\right)\to \psi_{b}\left(-x\right)$ in the scattering matrix element (7) [or its dressed version (8)] leads to a sign change in the GD, while the amplitude is left unchanged.

## V. CONCLUSION AND PERSPECTIVES

In summary, we have shown that using an APT ionization-seed in TDDFT calculations can produce experimentally relevant HHG spectra. Properly synchronized with the driving MIR field, the APT selects contributions from short quantum orbits, which in experimental measurements are filtered through macroscopic effect. Together with the ionization seed, a time window selecting the dipole/acceleration signal between two consecutive zeros of the electric field can be used to isolate the contribution from a single set of (short) recolliding orbits to the HHG spectra. This single half-cycle signal then provides a molecular-frame spectroscopic tool that discriminates between recollision from one end or the other of the oriented molecule.

We have applied this molecular-frame analysis of TCI in HHG spectra of CO_2_ and OCS, subjected to an MIR driving laser field. For the asymmetric case of OCS, we find systematic differences between recolliding from the O and S sides of the molecule, which are not compatible with field-free TCI geometric effects. Instead, by conceptually decomposing the bound wave-function between two components associated with the S and O ends of the molecule, we understand the differences between the two molecular-frame orientations in terms of transient polarization effects which reallocate electronic densities between the two “localized” centers. The original imbalance, in the field-free electronic structure, between the S and O ends is either compensated or worsened by the laser force. Depending on the instantaneous electric field at the time of recollision around the TCI, this leads to either sharpening or further extinction of the minimum. Conversely, the molecular-frame orientation angle can be used as a tuning parameter to control or scan this reshaping. Our analysis also stresses the importance of looking at trends by varying such control parameters. Indeed, individual spectra can be hard to read. On the other hand, here, angle-resolved and comparison of members of the OCR (R = O or R = S) family are keys to understanding transient TCI in our simulations.

The mapping between the relative electronic configuration around different groups of the molecule and the spectral properties of the TCI suggests that it provides a good landmark to study ultrafast, subcycle, electron dynamics in these systems. Such analyses, however, require a full characterization of the HHG signal, with both intensity and phase/group delay information.^17^ In the leading order and picturing the electron dynamics as a reallocation of densities between these localized fixed-in-space centers, one would expect the following: (i) the effective distance between the centers is encoded in the energy of the TCI. While it generally provides a poor prediction for that energy, the plane wave approximation gives the main mechanisms for understanding the orientation-angle dependence of the TCI energy. (ii) The relative amount of density between the centers is encoded in the spectral intensity, through the sharpness of the TCI feature, especially around destructive interference. (iii) The sign of the associated group-delay feature provides information on the orientation of that distribution, i.e., telling which center has more localized density. These concepts, together with the embedded time-frequency map of HHG, offer a promising framework to probe such ultrafast charge migration dynamics.
